# China's SMEs Developed Characteristics and Countermeasures in the Post-epidemic Era

**DOI:** 10.3389/fpsyg.2022.842646

**Published:** 2022-02-22

**Authors:** Wunhong Su, Xiaohan Guo, Yunxia Ling, Yi-Hao Fan

**Affiliations:** ^1^School of Accounting, Hangzhou Dianzi University, Hangzhou, China; ^2^Faculty of Business Administration, University of Macau, Taipa, Macau SAR, China

**Keywords:** L53, H11, H32, post-epidemic era, SMEs, policy support, sustainable development, China

## Abstract

Due to the COVID-19 outbreak, a series of chain reactions, like international trade breakdown, stock market collapse, and crude oil's collapse, have adversely affected the global economy, particularly small and medium-sized enterprises (SMEs). As a result, the Chinese government issued many fiscal and financial policies to support SMEs. This paper analyzes SMEs' coping methods and conceptual changes affected by the epidemic and distinguishes “victims” and “beneficiaries.” Subsequently, based on extensive international experience and local government experience, it provides effective suggestions for the Chinese government to deal with the post-epidemic era's economic changes, policy suggestions, and coping strategies for SMEs' short-term and long-term sustainable development.

## Introduction

The global spread of novel coronavirus has caused a huge economic shock that may exceed the global financial crisis of 2008 (Baker et al., 2020; Brown et al., 2020). China has borne the brunt, and its economic development has been greatly affected since China is the epicenter of the early outbreak. After the Spring Festival in 2020, all other enterprises in China will delay the resumption of work except those producing medical supplies. On March 29, the rate of small and medium-sized enterprises (SMEs) in China returning to work reached 76.8%, but the negative impact continued for a long time. In the first quarter, China's economy was severely affected by health incidents, with a year-on-year decline of 6.8%. But in the second quarter, our economy grew by 3.2%. Thus, China's epidemic prevention is very successful, only a few months.

Affected by the epidemic, Chinese enterprises cannot start normal operations and have minimal maintenance time. Among them, 67.1% can keep for 2 months, and 85.01% can maintain for 3 months at most. By the end of 2018, China has more than 30 million SMEs and more than 70 million individual industrial and commercial households, contributing more than 50% of the national tax revenue, more than 60% of the GDP, more than 70% of the technological innovation achievements and more than 80% of the labor force employment (Xinhua News Agency, 2019). Therefore, ensuring SMEs' survival and sustainable development to adapt to the economic situation under the epidemic will have a crucial impact on the healthy development of China's future economy and social stability.

The negative impact of the outbreak on SMEs is self-evident because of their vulnerability and need to sell to generate cash flow constantly. Compared with large enterprises, SMEs are at a disadvantage in the financing, innovation, and other aspects. Compared with large enterprises, SMEs have a relatively single way of financing, and the structure of the capital chain is too simple. The epidemic causes SMEs to shut down and the capital chain to break. Orders that cannot be fulfilled due to the outbreak also face specific financial compensation (Du, 2021).

For SMEs in the service industry, the source of capital is operating income. Once they stop operating, the source of capital will be exhausted. In the face of the shutdown of the epidemic, the office system of employees in enterprises has also been changed accordingly. Combining online and offline offices requires SMEs to have a complete online office system. Employees can freely allocate working time, and the firm can measure the performance of employees through the online work platform (Liu and Mou, 2021). Enterprise innovation is at the technical level and in the management mechanism and system innovation. Due to capital limitations, SMEs still need to improve their innovation (Xu, 2020). The development time of SMEs is not long, the effective credit duration is short, the solvency is weak, and the overall credit status is poor (Ding, 2020).

Faced with the situation of shutdown and production, sales difficulties, and investment decline, coupled with the unabated pressure of rigid expenditure such as rent and interest, combined with other factors such as the expiry of accounts payable at the end of the year, the survival pressure of SMEs is further intensified. As a result, according to the data of the China Association of Small and Medium-Sized Enterprises, 2020, the Small and Medium-sized Enterprises Development Index (SMEDI) in February was 76.4, a sharp drop of 16.2 points compared with the previous month and a record low since the survey was started in the second quarter of 2010.

Under normal circumstances, the life cycle of SMEs in China is only three and a half years. Still, under the influence of force majeure such as COVID19, the mortality of SMEs may increase by 50% (Lu and Xu, 2020). The Chinese government has promptly issued inclusive fiscal and financial policies to support SMEs' sustainable development to mitigate the epidemic's impact on SMEs, a vital part of the economy. This paper aims to analyze the epidemic's impact on SMEs in China, mainly focusing on SMEs' development status and characteristics in the post-epidemic era, and then put forward policy suggestions to support the healthy development of SMEs and lead the economic recovery (Table 1).

**Table 1:** Quarterly quarter of China's SMEDI in the fourth quarter of 2020 and 2019.

**Divisions**	**2020 Q4**	**2020 Q3**	**Gains**	**2020 Q2**	**2020 Q1**	**Gains**	**2019 Q4**	**2019 Q3**	**Gains**	**2019 Q2**	**2019 Q1**	**Gains**
The total index	87.0	86.8	0.2	85.5	82.0	3.5	92.7	92.8	0.1	92.8	93.1	0.3
Industrial	87.8	87.6	0.2	86.3	82.4	3.9	92.5	92.6	0.1	92.4	92.7	0.3
The construction industry	91.5	91.2	0.3	89.6	85.4	4.1	97.0	97.2	0.2	97.1	97.3	0.2
The transportation	77.0	76.7	0.3	75.6	73.1	2.5	85.7	85.7	0.0	85.8	85.9	0.1
The real estate	94.6	94.8	0.2	93.2	89.5	3.8	98.8	99.0	0.2	99.2	99.5	0.3
Wholesale retail	87.1	86.9	0.2	86.3	82.8	3.5	92.6	92.7	0.1	93.0	93.1	0.1
Social services	88.1	87.7	0.4	86.2	83.2	2.9	94.9	95.2	0.3	95.5	95.8	0.3
Information transmission software industry	83.0	82.9	0.1	82.4	81.3	1.1	92.3	92.4	0.1	92.7	92.9	0.2
Accommodation and catering industry	66.6	66.2	0.4	64.8	64.6	0.2	77.9	78.1	0.2	78.0	78.1	0.1

## Literature Review

As an external impact of force majeure, COVID-19 will significantly affect the book value of enterprises. However, the intrinsic value of enterprises following random Brownian motion may increase during the epidemic period (Wang et al., 2020). When the book value of the enterprise is <0, the risk of bankruptcy will make the enterprise face the danger of the disappearance of intrinsic value. To prevent the internal value of enterprises from being threatened due to the low book value, the government needs to actively introduce supportive policies in the critical period such as the epidemic to help SMEs to tide over the operating difficulties and avoid the crisis of macroeconomic development and social stability caused by a large number of failures of SMEs.

Salimzadeh et al. (2013) constructed the sustainable development model of SMEs. They proposed that the government is one of the main external factors affecting the healthy development of SMEs. Bouazza (2015) also believe that national policies, legal systems, and supportive policies have a non-negligible impact on the growth of SMEs. All these documents prove that the government does have an important influence on the healthy development of SMEs. Even in a period of stable economic growth, the government can recognize this and provide specific policy guarantees for the healthy development of SMEs (OECD., 2009).

However, some government policies and behaviors and a bad external business environment will also have a negative impact on the sustainable development of SMEs. In an IFC study, responses from 45,000 enterprises in developing countries revealed that corporate sustainability's core factors include heavy tax burdens, complex legal systems, and cumbersome administrative procedures. The World Bank (2020) survey also indicated that a complex tax system, low trust in the judicial system, and rent-seeking demand for access to public services are the main obstacles facing SMEs. All these negative effects above come from the inappropriate policies of the government. Therefore, to promote SMEs' healthy and sustainable development, the government must establish an efficient legal and policy system and a reasonable tax system to reduce the management cost of SMEs (Harvie and Lee, 2005).

Compared with large enterprises, SMEs have the worse anti-risk ability under force majeure (Liu, 2009). The subprime crisis in the second half of 2007 led to the bankruptcy of about 67,000 SMEs in China (Lu and Xu, 2020). According to the data of China's Association of Small and Medium Enterprises, the development index of small and medium enterprises in the first and second quarters of this year dropped by 11.1 points and 7.3 points, respectively, compared with last year, indicating that the healthy development of small and medium enterprises was seriously hindered under the epidemic.

Therefore, SMEs have a low ability to fight against the epidemic and other force majeure and face practical problems such as insufficient liquidity and lack of funds. Moreover, the epidemic tests the modernization of China's governance system and capacity. Therefore, formulating appropriate policies is very important, related to whether SMEs can tide over the difficulties and long-term development.

## Characteristics of the Impact of the Epidemic on SMES

### Liquidity Crisis

SMEs often face serious liquidity problems in the face of the epidemic. Due to incomplete data in the fourth quarter of 2020, listed firms' operating income and profit on the small and medium-sized board from the first quarter to the third quarter of 2019 and from the first quarter to the third quarter of 2020 were selected. As a result, the operating income of small enterprises in the first quarter of 2020 decreased as high as 52.944 billion yuan compared with that in the first quarter of 2019. Operating income as an important source of enterprise funds, the substantial decline of the transaction amount has endangered the liquidity of SMEs.

The turnover only considers the cash inflow from operating activities but the cash outflow caused by operating costs. Therefore, this paper chooses to use the profit and loss index to reflect the cash income status of enterprises more accurately. The total profits of SMEs in the first quarter of 2020 decreased by 3.82 billion yuan compared with that in the first quarter of 2019. Due to the Spring Festival in the first quarter, all enterprises were in production and suspension combined with the epidemic's impact. As a result, the profits of SMEs decreased in the first quarter of 2020, while in the second and third quarters, due to the resumption of work and production, effective epidemic prevention and control, and preferential policies for SMEs, they recovered to the level before the epidemic.

In 2020, the various regional departments seriously implemented the central decision-making to deploy, as a whole, the epidemic prevention, and control and economic and social development, solid foundation to do a good job of “six stability,” the full implementation of “six protect” task, restore order to the production and living actively, effort to safeguard the people's livelihood product supply, improving market supply and demand relations, PPI from the low rise, basic stable price movements all the year-round. PPI fell first and then rose.

In 2020, the PPI will drop by 1.8%, 1.5 points more than the previous year. On a month-by-month basis, due to the epidemic and other factors at the beginning of the year, the demand for industrial products was weak. Since February, PPI has been in a declining range month-on-month and year-on-year. However, as China's epidemic prevention and control situation has continued to improve, industrial production has recovered steadily, investment in infrastructure and real estate has continued to boost, and some international commodity prices have fluctuated upwards, the PPI has stopped falling month-on-month and turned higher since June, and the year-on-year decline has steadily narrowed. In December, the PPI rose by 1.1% month-on-month. Thus, the PPI is roughly close to the level before the outbreak hit in absolute terms.

Monthly CPI is higher than before and lower than before. In 2020, the CPI will rise by 2.5%, 0.4 points lower than the previous year. Minimal look, compared with the overall high before the downward trend. In the first 2 months of this year, due to the combined effects of COVID-19 and the Spring Festival, the prices of pork and other food products rose rapidly, driving the CPI up by 3.64 and 3.68%, respectively. As the epidemic prevention and control situation continues to improve, pig production capacity continues to recover, and various measures to ensure supply and stabilize prices continue to work, CPI inflation fell from March.

In June, the increase expanded slightly due to extreme weather, such as high temperatures and rainfall. However, CPI continued to fall from August. IN DECEMBER, the CPI rose 0.7%, driven by cold weather, increased demand, and rising costs. Food prices rose sharply. In 2020, food prices rose by 10.6%, 1.4 percentage points higher than the previous year, influencing the CPI rise by about 2.20 percentage points and being the main factor driving the CPI rise.

As business activities are restricted, SMEs rely more on internal funds and external financing. Internal sources of capital are crucial for start-ups and SMEs (Robb and Robinson, 2014). However, SMEs' spare funds and precautionary savings are quite limited, and they will be exhausted in a short time. Therefore, external funding is crucial (Brown and Lee, 2019). Especially under an economic downturn such as the global financial crisis, it will be more difficult for SMEs to obtain bank financing (Cowling et al., 2012; Lee et al., 2015; Demirguc-Kunt et al., 2020). This is particularly reflected in times of crisis when uncertainty increases, which is associated with a rapid rise in borrowers' pessimism about future economic conditions (Cowling et al., 2016).

In addition to bank financing, many innovative SMEs are also widely dependent on venture capital and business angel investment (Brown and Lee, 2019). However, drawing on data from the UK, this type of financing fell by 30–40% in the first quarter of 2020 (Brown et al., 2020). In the face of internal and external financing difficulties, SMEs will appear obvious shortage of funds. According to the “Joint Investigation of 995 Small and Medium Enterprises by Tsinghua University and Peking University” published by China-EU Business Review, 85.01% of SMEs can only maintain their funds for 3 months at most, 34% of them can only maintain their funds for 1 month, 33.1% can maintain their funds for 2 months, and the only 9.96% can maintain their funds for more than 6 months.

In the case of insufficient funds, SMEs unable to pay their current liabilities tend to choose to minimize the cost, resulting in SMEs reducing their investment in activities conducive to the growth of enterprises, such as innovation, capital, and international trade, and ultimately inhibiting the healthy development of SMEs (Brown et al., 2019a,b). Low prices and small profits may save the firm for a while, but eventually, it will be replaced by constantly replacing technology products until it loses product market share. Faced with severe liquidity problems, SMEs may seek non-traditional sources of funds, such as high-interest loans, to pay off debts and maintain development (Brown et al., 2019a,b). However, if the cost of loans is too high, it will also hinder the long-term sustainability of SMEs. Therefore, SMEs are facing a severe liquidity crisis and are in urgent need of government support.

### Heterogeneity of SMEs

The different SMEs are affected differently by the epidemic. Even in the negative impact of the economic crisis, some firms benefit. However, at the same time, certain types of SMEs are at greater risk than others. A classic example is losing market share to online retailers that offer in-home delivery. In addition, traditional retail and restaurant businesses shut down due to blockades or a decline in customers. Therefore, SMEs' heterogeneous performance is classified as beneficiaries, general beneficiaries, irrelevant, general victims, and victims.

The epidemic victims are SMEs facing serious operational difficulties and bankruptcy dilemmas under the epidemic's impact. This negative impact mainly comes from the vulnerability of SMEs, primarily in the form of single access to financing and a simple capital chain. On the other hand, the beneficiaries are the “winners” in this crisis, whose benefits are expressed in the unique business model and industry characteristics that bring them funds to maintain a normal or even profitable business situation. At this time, identifying the most vulnerable enterprises to the negative impact of external shocks and issuing targeted policies will be more conducive to ensuring the sustainable development of SMEs.

#### SMEs That Are More Affected by the Epidemic—Victim Groups

In terms of the types of SMEs, micro-enterprises and start-ups with low production capacity are typical “victims” because they are facing serious financing difficulties and business difficulties and may go bankrupt at any time. For innovative SMEs, financing constraints can make them “discourage” borrowers and thus fall into the category of “general victims.” In terms of regional distribution, SMEs located in remote areas face greater challenges in financing and fall into the “victim” category. In contrast, SMEs fall into the “general victim” category in other areas. In terms of industry, localized service-oriented SMEs such as accommodation, catering, and tourism are most vulnerable and fall into the “victim” category. In contrast, other SMEs fall into the “general victim” category.

First, micro-enterprises with the lowest production capacity are the most vulnerable. They are the firms with the weakest cash reserves and the lowest access to external debt financing. Therefore, it is difficult for them to alleviate the liquidity crisis under the epidemic by operating independently. Importantly, studies have also shown that they are often reluctant to borrow money from banks for fear of being rejected (Cowling et al., 2012), making micro-enterprises suffer more severe liquidity problems under the epidemic. In addition, in the process of traditional offline enterprises being squeezed by online enterprises, the least productive enterprises are also the most vulnerable to being eliminated (Haldane, 2018).

Start-ups are also vulnerable to outbreaks (Kuckertz et al., 2020). Start-ups face funding difficulties caused by the outbreak just when they most need outside funding to get started. Of course, some traditional start-ups can rely solely on their capital, but the more innovative and capital-intensive start-ups are more likely to rely on loans for funding. During the outbreak, credit has contracted dramatically, making it harder for these start-ups to get financing. The shortage of capital is so significant that start-ups will be at risk of not operating normally.

In addition, start-up enterprises initiated during recessions start small and continue to grow even after macroeconomic conditions gradually recover. In the last week of March, new business applications in the United States fell 40% from a year earlier, according to the US Census Bureau, a contraction even worse than the Great Recession. Start-ups being hit by the epidemic means fewer successful entrepreneurs and a lower quality of start-ups, which could also negatively impact the level of innovation in China's economy in the future.

For innovative SMEs, the funding problem is very serious (Lee et al., 2015). Banks are often reluctant to lend money since innovative SMEs often have limited collateral, intangible intellectual property rights, and unstable cash flow. As a result, it is difficult for such enterprises to obtain bank financing (Lee and Brown, 2017). At the same time, innovative firms face higher financing constraints and may self-restrict capital, thus becoming “discouraged” borrowers (Brown et al., 2018). Compared with other SMEs, they are more important for industrial innovation. Therefore, reducing the financing constraints of innovative enterprises is an important policy objective in times of economic recession, which can help these firms withstand risks and better accept strategic investment from society and the government.

There are also differences in the ability of SMEs to obtain capital in different regions. For example, from the perspective of urban-rural distribution, SMEs located in townships, county-level cities, and suburbs may be more vulnerable to the epidemic's impact. On the other hand, studies have shown that SMEs located in rural and remote areas will face greater challenges accessing bank financing (Lee and Brown, 2017). The reason is that there are fewer bank branches in remote areas and less competition among banks, which leads to unfavorable loan conditions for SMEs. At the same time, SMEs lack the opportunity to establish a good relationship with banks, limiting the possibility of increasing loans.

Moreover, in the wake of the global epidemic crisis, many banks have drastically reduced their face-to-face banking services, further exacerbating the financing problems of rural SMEs. From the provincial and municipal distribution perspective, enterprises in Hubei and Wuhan, the epicenter of the COVID-19, bear the brunt. From the perspective of macroeconomic data, according to the Hubei Provincial Bureau of Statistics. (2020), the GDP of Hubei Province in the first quarter of 2020 was 637.935 billion yuan, down 39.2% year on year. In addition, the added value of the primary industry decreased by 25.3%, the secondary industry decreased by 48.2%, and the tertiary industry decreased by 33.3%. Such a bad macroeconomic environment hindered the development of SMEs.

On the industrial side, localized service-oriented SMEs are the most vulnerable. SMEs' economic activity in February and March was only 10.2 and 11.8% of the same period in 2019, due to delays in major schools and training institutions. Moreover, as residents cut back on going out, partying, and other activities, the recovery of economic activity in the accommodation and restaurant sectors in February and March 2020 was only 12.8 and 23.5% compared with the same period last year.

With improved disease control, the second half of 2020, as the “six steadies” “six protect” policy gradually pay off, landing the epidemic prevention and control as a whole. Furthermore, positive progress was made in economic and social development work. The economy continues steadily since the second quarter picks up momentum, main indicators continue to improve on both ends of the supply and demand, market main body dynamic enhanced obviously, economy stabilizes positive posture, basic establishment, and further consolidated recovery.

At the same time, the current international environment is still complex and grim, the global epidemic is still spreading, external demand is shrinking, protectionism and unilateralism are prevalent in some countries, and globalization is facing a headwind. In addition, there is growing pressure on enterprises to maintain stable employment and ensure people's well-being in China. Therefore, the foundation for a steady economic recovery still needs to be strengthened.

SMEs still face many difficulties in production and operation, heavy burdens, financing difficulties, rising costs, insufficient orders, and other problems, and operating pressure continues to increase. Since July, the sub-sector index and sub-index have been six rises, 1 level, and one decline. Among the surveyed SMEs, 93.55% have resumed work. The rate of resuming work in the wholesale and retail sectors was 96.67%. The worst was the hotel and restaurant industry, with a return rate of 89.47%. The indices for industry, construction, real estate, wholesale and retail trade, information transmission and computer software, and accommodation and catering rose 0.3, 0.4, 0.4, 0.3, 0.5, and 0.3 points respectively compared with the previous month.

Another industry that has suffered a huge impact is tourism. Due to the need for security and epidemic prevention and control, many tourists have reduced their travel plans, and tourism-related enterprises, especially SMEs, have been greatly affected. For example, Baicheng Travel, which was once rated as a “5A-class travel agency,” started bankruptcy liquidation on February 28, 2020, which was once seen by Chinese media as the beginning of a wave of bankruptcies in the tourism industry. In addition, the outbreak has severely affected industries and sectors such as sports, cultural entertainment, and construction.

These SMEs, particularly vulnerable to the epidemic, are also experimenting with various self-help forms to minimize the use of loans to get through the fallow period and reduce operating costs. Responsive measures include extending executive pay plans, reaching agreements with suppliers to rent equipment rather than buy it, and running businesses with personal savings. However, under the severe impact of the epidemic and other crises, this short-term form of financial self-help is often difficult to solve the serious capital shortage of SMEs. As a result, many SMEs face the risk of bankruptcy in the long run.

#### Types of SMEs That Are Less Affected by the Epidemic—Beneficiary Groups

For now, the winners from this crisis are more likely to be high-tech, digital industries. They are widely engaged in life sciences, medical equipment, online delivery, software services, and other businesses. Moreover, they are highly resilient in the face of epidemics because of their online business model. In their research, Brown and Lee (2019) found that high-growth enterprises can adjust and grow rapidly even in times of crisis. One important reason is that some firms receive venture capital or angel financing, which gives them enough capital to alleviate liquidity problems, belonging to the “general beneficiaries” category. Other businesses are less vulnerable because of certain industry characteristics. For example, firms in the computer game industry can still get money from customers to develop new games. During the epidemic, long hours at home increase the demand for games, belonging to typical “beneficiaries” of the epidemic. Other firms that have thrived in the epidemic may have benefited from good business models, sufficient reserves of capital, and good management teams. There is no shortage of ordinary SMEs adopting new business models, restructuring the organization, and flourishing. For example, a traditional local restaurant can become a successful local ready-to-eat food manufacturer if it changes its business.

#### Types of SMEs Not Affected by the Epidemic

In addition to the victim and beneficiary groups mentioned above, some industries are unaffected by the epidemic or have minimal impact (Wu and Liu, 2021). First, the epidemic does not affect the food and some necessities industries because of their small demand and price elasticity. Second, the domestic new energy manufacturing industry is virtually unaffected by the epidemic due to its high level of automation. For example, the intelligent manufacturing workshop has a fully enclosed intelligent manufacturing automation production line consisting of automatic gripping robotic arms and intelligent transportation robots. Therefore, production is unaffected by the contagiousness of the epidemic.

The technical support of the automation industry belongs to the information technology industry. The information technology industry has a strong seasonality, the first quarter in the order bidding period, rather than the scale of the implementation of the period. Therefore, although the performance accounted for a small percentage, the impact on the whole year is limited. In addition, some of the short-term suppressed demand is released after the epidemic's end, and the overall performance is adjusted in time but is not be affected too much in total.

In addition to automation and information technology industries, industries affected by the epidemic include TMT, biologics, and medical devices in pharmaceuticals, new energy, semiconductors, military industries. The boom is relatively high and continues throughout the year. Although the epidemic affects short-term industry ups and downs, the epidemic does not change the long-term trends in the industry.

## Analysis of China's Existing Policies

### Summary of Existing Policies

Since the outbreak of COVID-19, Chinese governments at all levels have conscientiously implemented the decisions and arrangements of the CPC Central Committee and the State Council and introduced many programmatic or specific policies to support local prevention and control of the epidemic. As the affected extent of the epidemic varies from place to place, the policies issued by local governments also vary. The following is a summary of the main policies of the central government and some representative local policies (Tables 2, 3).

**Table 2:** Policies at the national level.

2020.1.26	China Banking and Insurance Regulatory Commission	Notice on strengthening financial services of banking and insurance industry to cooperate with the prevention and control of novel coronavirus infected pneumonia
2020.1.30	State Administration of Taxation	“Notice on optimizing tax payment services and cooperate with the prevention and control of novel coronavirus infected pneumonia”
2020.1.30	People Club Bureau	Notice of the Ministry of Human Resources and Social Security on further improving the prevention and control of novel coronavirus infected pneumonia
2020.2.1	The Ministry of Finance	Notice on duty-free policy of imported materials for prevention and control of novel coronavirus infected pneumonia
2020.2.1	General Administration for Market Supervision	Notice of the General Office of the State Administration for Market Supervision on the Registration of Market Subjects during the prevention and control of novel coronavirus infection pneumonia

**Table 3:** Policies at the local level.

2020.2.3	The Beijing Municipal	Several measures of the General Office of the Beijing Municipal People's Government on further supporting the prevention and control of novel coronavirus infected pneumonia
2020.2.3	Shanghai	Notice of Shanghai Municipal Bureau of Human Resources and Social Security on the Social Insurance Administration of this Municipality during the prevention and control period of novel coronavirus infected pneumonia
2020.2.4	Chongqing	The General Office of Chongqing Municipal People's Government on the response to novel coronavirus pneumonia epidemic to support SMEs to tide over the difficulties together
2020.2.5	Zhejiang province	18 suggestions of Zhejiang Provincial leading group for the prevention and control of novel coronavirus infected pneumonia on SMEs to survive a hard time
2020.2.6	Guangdong province	The People's Government of Guangdong Province issued a notice on several policies and measures to support the resumption of work and production of enterprises in response to the novel coronavirus pneumonia epidemic

#### Increase Credit Support

On January 31, 2020, China Banking and Insurance Regulatory Commission, the People's Bank of China, and other five departments issued the Notice on Further Strengthening Financial Support for the Prevention and Control of Novel Coronavirus Pneumonia Epidemic, requiring the People's Bank of China to continue to strengthen expected guidance, maintain reasonable and abundant liquidity, and increase credit support for the epidemic prevention and control related fields. In addition, differentiated and preferential financial services will also be provided to regions, industries, and enterprises greatly affected by the epidemic. Particularly, the circular calls for financial institutions to strengthen service capacity building by focusing on internal resource allocation, incentive, and assessment arrangements. Besides this, continue to increase support for small and micro enterprises and private enterprises, maintain loan growth, earnestly implement the requirements for reducing total financing costs, and improve medium—and long-term loans to the manufacturing sector.

#### Tax Reduction for SMEs

Cost subsidy has become the biggest appeal of SMEs to the government. According to the questionnaire, 50.2% of enterprises hope the government provides subsidies or exemptions in social security, rent, employee salary, and other cost expenditures. For example, from February to June 2020, the phased exemption of small and medium-sized enterprise pension, unemployment, industrial injury insurance unit payment. In addition, from February to June 2020, in areas where the accumulated balance of the employee health insurance pooling fund can be paid for months greater than 6 months, the contribution of the employee health insurance unit can be halved, and the phased fee reduction policy can continue to be implemented until April 30, 2021.

Enterprises affected by the epidemic that cannot pay social insurance premiums on time will be allowed to postpone payment until 3 months after the epidemic's end, during which no late fee will be charged. In addition, enterprises with serious production difficulties affected by the epidemic may apply for postponement of payment of social insurance premiums. The execution period for postponement of payment is within 2020. In principle, the deferment period shall not exceed 6 months, and late fees shall be exempted during the deferment period. In addition, private leasing enterprises with state-owned assets severely affected by the epidemic and unable to operate normally will be exempted from the first month's rent and halved from the second and third months 'rent to ensure reduced or reduced rent actual operating tenants.

The eligible state-owned units will be encouraged to appropriately extend the time limit for rent remission or exemption. During the epidemic prevention and control period, enterprises will be allowed to delay filing and pay taxes. Enterprises that meet the conditions for postponing tax payment shall be allowed to extend the tax payment period by no more than 3 months. For enterprises with difficulties paying taxes, property tax and urban land use tax will be reduced or exempted by the law. Furthermore, reducing the VAT rate of small-scale taxpayers from 3 to 1% from March 1, 2020, to May 31, 2020, will be implemented.

In Guizhou and other places, SMEs with difficulties in production and operation affected by the epidemic can reduce their housing provident fund contribution ratio to at least 5% as stipulated by the state, and eligible SMEs can postpone their contribution for up to 1 year. In addition, in Chongqing, Guizhou, and Fujian provinces, local governments have also introduced a 2-month exemption from property tax and urban land use tax for SMEs that have been heavily affected by the epidemic, reducing taxes and burdens for SMEs.

According to the public information released by the Central People's Government of China (2020a,b), statistics of the State-owned Assets Supervision and Administration Commission (SASAC); the central enterprises have taken the initiative to reduce costs and burdens for small and micro enterprises in the first half of the year, with a total of more than 4 billion yuan of rent reduction and exemption.

#### Reduce the Interest Rate of Bank Loans

Since 2020, the People's Bank of China has adopted reform measures to smooth the transmission of monetary policy and continuously promoted the reform of loan market quoted interest rate (LPR), which has significantly reduced the financing cost of enterprises. Under the government's guidance, commercial banks have offered more favorable loan rates to SMEs. For example, CCB's interest rate on loans to SMEs in Hubei dropped by 0.5% The Bank of Communications lowered the lending rate by 0.5% for enterprises related to epidemic prevention and further reduced the lending rate for some key enterprises.

The Agricultural Bank of China has also offered preferential loan rates to key industries and enterprises related to epidemic prevention. Financial institutions should not blindly withdraw loans, cut off loans or squeeze loans. Due to the epidemic, small and micro enterprises facing repayment difficulties should extend the term, renew loans, reduce unpaid interest and other assistance, and properly arrange repayment plans until the “first-level response” is removed.

In general, with the joint efforts of many parties, the financing of small and micro enterprises in 2020 has achieved a significant effect of “quantity increase, price decrease, and scope expansion.” By the end of 2020, the balance of small and microloans of Pratt and Whitney was 15.1 trillion yuan, up 30.3% year on year. In December 2020, the interest rate of newly issued Pratt and Whitney small and microloans was 5.08%, down 0.8 percentage points from the same period in 2019. In 2020, a total of 32.28 million small and micro businesses were supported, up 19.4% year on year.

#### Allow the Loan to Defer Repayment of Principal and Interest

On March 1, 2020, silver was issued by the Ministry of Finance on a temporary extension to micro, small and medium enterprises loan servicing notice, put forward by the principle of marketization, government by law, to comply with conditions, liquidity temporary difficulties of small and medium-sized enterprise loan, giving a brief delay servicing arrangements, the longest can be extended to June 30, 2020.

In Hubei province, the worst-hit area, the circular also requires banking financial institutions to provide Hubei with a special credit scale, implement preferential internal fund transfer pricing, and strive to reduce the total financing cost of inclusive small and micro businesses by more than one percentage point from the average level of the previous year in 2020.

#### Keep Employment of SMEs Stable

According to Government of China (2020a,b), the State Council issued the “Opinions on the Implementation of the Measures for Strengthening and Stabilizing Employment in Responding to the Influencing of COVID-19” in March, which proposed to cancel the unreasonable approval that restricts the resumption of work and production and improves the standard for the return of unemployment insurance for small, medium and micro-enterprises. Furthermore, in terms of entrepreneurship, the government will also optimize the environment for self-employment, deepen the reform of “separation of licenses and licenses,” expand the coverage of guaranteed loans for entrepreneurship, and provide policy support to venture capital enterprises.

#### Increase Government Procurement Support

Many local governments have explicitly proposed government procurement for SMEs to encourage production. For example, in February 2020, the Chengdu municipal government issued 20 policies and measures to effectively deal with the epidemic and stabilize economic operation, which mentioned that the government should take measures such as reservation share, preferential evaluation, and eliminating threshold, and strive to make the proportion of goods and services purchased by SMEs reach more than 80%. In addition, establish and improve the advance payment system, and encourage purchasing units to advance a certain percentage of the contract price to suppliers winning the bid.

### Policy Analysis

The preceding discussion indicates that certain SMEs are vulnerable to the epidemic, reducing their future viability and growth potential. In the following part, the relevant support policies issued by the Chinese government and the current situation in China will be analyzed to provide suggestions for the government to introduce more targeted policies and measures to reduce the negative impact of the epidemic crisis on SMEs.

#### Analysis of Policy Implementation Strategies

Government policies can be divided into long-term and short-term policies from the duration of benefits. In the short term, the government needs to act quickly to ensure that SMEs can continue to operate, ease liquidity constraints for SMEs, and ensure employment stability (OECD., 2020). The Chinese government is equally committed to this. For example, in terms of employment, the State Council has proposed that small, medium, and micro-enterprises that do not lay off employees or fewer layoff employees can refund up to 100% of the unemployment insurance premiums paid by the enterprises and their employees in the previous year.

In terms of financing, the government proposed to strengthen credit support for manufacturing, small and micro enterprises, private enterprises, and other key areas, strengthen the construction of banking services, maintain the growth of loans, earnestly implement the requirements for reducing the pressure of total financing costs, and increase the supply of medium—and long-term loans to the manufacturing sector.

Accordingly, policies have also been introduced to allow small, medium, and micro enterprises temporarily facing liquidity difficulties to delay interest payment and to increase the reloan and rediscount quota of 500 billion yuan for small and medium-sized banks to increase credit support to small, medium, and micro-enterprises, and to reduce the reloan interest rate for agriculture and small by 0.25 percentage points to 2.5% (China.gov.cn., 2020).

But in the long run, policy support must be different from the short-term model, that is, more to promote enterprise growth and transformation and upgrading, rather than to support enterprise survival as the goal. This requires the government to have a long-term outlook on development, supporting entrepreneurship, promoting innovation, supporting high-growth enterprises, helping enterprises develop internationally, which will be the core goal of the government to help SMEs gradually recover.

#### Policy Risk Forecast Analysis

While the implementation strategy after policy enactment is important, it is equally important to anticipate policy risks during the policy development process (Pal et al., 2020). Therefore, this study considers a series of social and economic problems that the new policy may cause in proposing policy recommendations and has made some suggestions to improve the situation to avoid the risks faced by the implementation of the new policy. The policy proposed for SMEs seems a single risk factor. Still, after big data analysis, policy implementation can directly or indirectly cause other risks, such as policy changes that can lead to business model risks, regional environmental risks, and economic risks.

First, the implementation of the new policy may impact the business model of SMEs. Since the business model of online operation is full of operational flexibility in the face of the epidemic, the business model of SMEs heavily focuses on the online operation. On the one hand, the business model of online operation enables enterprises to achieve the best integration of human, material, and financial resources to improve their business dilemma under limited conditions (Zhang et al., 2015). However, on the other hand, a single business model of online operation poses some problems (Xing, 2021), such as low efficiency of supply chain management, inefficient management of operation teams, and low application of new technology tools.

In response to the above problems, the government must firstly build an efficient supply chain system, use modern information analysis technology, plan paths to control inventory, accurately predict consumer demand to optimize production and reduce costs while meeting the decentralization of consumer demand and customer timeliness requirements, to create an intelligent supply chain system that makes information flow, capital flow and logistics closely linked, and improve operational efficiency. Secondly, the training of composite professionals. In the face of the new situation, in 2021, the state has increased the training of big data and artificial intelligence composite professional talents, putting forward the opinion of “artificial intelligence + X.” At present, 214 universities have been approved to add artificial intelligence undergraduate majors and simultaneously require basic theory and technical applications to promote the development of artificial intelligence technology. Furthermore, colleges and universities should align with society in terms of training objectives, curriculum, faculty deployment, practical teaching design to cultivate composite talents that meet the needs of society to serve society and economic development.

Finally, a complete system of laws and regulations should be improved. In addition, the government should introduce related support policies to guide and regulate the content of online business, such as ensuring that the website's pages are simple to operate and can be operated by personnel of different cultural levels, while specifically setting up a feedback platform for employees' opinions to strengthen teamwork, and introducing related policies to protect enterprises with relatively weak comparative advantages and avoid the phenomenon of a dominant industry to ensure a fair and orderly market.

Second, regional policy risk. The post-epidemic era is still challenged by short-duration and sub-regional outbreaks of the epidemic. Based on an improved multiregional computable general equilibrium analysis model (TERM-China), Wu et al. (2021) simulate the impact of the COVID-19 outbreak on the Chinese economic system and industrial sectors in a partitioned and hierarchical manner, as well as the degree of economic and industrial recovery after different adaptation measures. The results show that the change in aggregate and pattern of consumer demand from the epidemic shock lead to a short-term decline in consumption, investment, employment, and an increase in prices. Delayed resumption of work and traffic management control about labor inputs, higher labor wages, higher production costs, and lower supply for firms. This also suggests that introducing new policies could lead to a “one-size-fits-all” problem across the country, creating regional policy risks. To address such risks, the government should increase public health care investment to control the epidemic while maintaining the principle of “top-down” precision in implementing both epidemic prevention and control and economic recovery.

Key industries affected by the epidemic can be identified at the national level. In the short term, prudent and accommodative monetary policy can reduce business operating costs and increase the counter-cyclical adjustment of fiscal policy to expand the scale of fiscal spending. At the regional level, the epidemic's impact on the consumption channel is more rapid and direct, which in turn is transmitted to the upstream manufacturing industry. Therefore, while paying attention to the direct impact of the epidemic on SMEs, the government needs to pay attention to the indirect industry of supply chain transmission. At the enterprise level, practical assistance should be provided based on actual production needs, and deregulation should be accompanied by improved regulatory efficiency to stabilize employment and protect people's livelihood.

Finally, the implementation of new policies may generate certain economic risks. Economic risk refers to the risk caused by the uncertainty of economic policies on living standards and market supply due to macroeconomic environment changes (Yu et al., 2011). There is great uncertainty in the future development of the epidemic, so the proposed new policies such as government purchase and financial support are bound to lead to dynamic fluctuations and uncertainties in the market, thus triggering economic risks. To address such risks in advance, the government should increase counter-cyclical regulation as soon as possible to stabilize the economy. Macroeconomic policies are demand-side cyclical regulation tools that, if used properly, can scald economic fluctuations and improve welfare (Gao et al., 2017). However, since they are cyclical regulatory instruments, they should neither be overused nor relied on for long periods. Supporting sustainable economic growth requires relying on structural reforms on the supply side. Therefore, the government should integrate with supply-side reforms while introducing new policies to avoid losing sight of the other.

## Policy Recommendations

This study's analysis is based on the “victim group” and “beneficiary group” under the epidemic impact. The policy's objective is to alleviate the difficulty of most SMEs, i.e., the “victims” and the “general victims.” According to the Nash equilibrium in game theory, the “victim group” and the “beneficiary group” must form a non-cooperative game state for both sides to gain greater benefits. Therefore, the proposed new policy does not negatively impact the “beneficiaries” while helping the SMEs in the “victim group” category but promotes a better situation. Given the difficulties faced by most SMEs, this study proposes both short-term and long-term policies.

### Short-Term Policy Recommendations

This paper puts forward a series of key short-term policy recommendations to directly alleviate the epidemic's impact on SMEs in China, ensure their survival, and promote SMEs' healthy and sustainable development. Here are seven suggestions from this article.

Firstly, it should set up an employment guarantee fund for SMEs to attract jobs. A one-time subsidy will be given to SMEs that resume production during the epidemic period. In addition, incentives will be given to SMEs that actively expand employment absorption. At the same time, financial support and conditional technical guidance will be provided for individual entrepreneurship and innovation. With leading enterprises in the industry as the core, such employment guarantee fund for SMEs can fully explore and utilize financing resources of industrial chain and commercial ecological operators, drive more enterprises in industries or subdivided industries to resume operation, and restore the ability of SMEs to absorb employment. Listed firms and other large enterprises should be excluded from the SME Employment Guaranteed Scheme (except Hubei Province) to ensure that these large enterprises do not encode government funds for SME employment recovery.

Secondly, it should fully play the employment-absorbing capacity of the fast-growing SMEs in the epidemic. Policies should be put in place to connect these fast-growing SMEs and ensure that they can better absorb employment. For example, some economists argue for hiring subsidies to encourage firms to engage more in times of crisis.

Thirdly, encourage enterprises to take the initiative to raise financial needs. As mentioned above, the financing difficulties of SMEs not only exist on the supply side but also on the demand side. Many enterprises refuse to actively seek external financing opportunities for fear of financing failure, resulting in a serious shortage of corporate capital. According to the study, capital investment by SMEs will fall significantly under the outbreak (Herbane, 2010; Doshi et al., 2018). At this time, the Chinese government should assume the main responsibility for matching supply and demand, publicize various means to help SMEs get funding, and encourage them to take the initiative to get financing.

Fourthly, strengthen financial support for SMEs. In addition to lowering the loan threshold for SMEs by state-owned banks, various social banks and other financing organizations can also be encouraged and rewarded to open the green light for SMEs and launch the capital pool of private enterprises to promote the economic recovery of SMEs. Furthermore, as Brown (2020) suggested for the National Investment Bank of Scotland, productivity clauses could be included in loans to SMEs to encourage them to provide training programs such as skills education for their staff to support the vulnerable groups in these epidemics energize future corporate innovation.

Another approach is offering free financial advice to small and medium-sized firms from different funding sources, particularly those too small to understand complex financial products fully. Increasing SMEs' access to external financing information and improving their understanding will help them obtain financing suitable for themselves (Wilson, 2015).

Fifthly, promote the combination of SMEs and online business models. The Chinese government has provided 500 billion yuan of additional loan funds and other preferential financing policies for SMEs. This is currently the practice in most developed countries (OECD., 2020). However, according to the analysis data of the National Bureau of Statistics (2020), in February 2020, affected by the epidemic, the purchasing managers' index (PMI) of China's manufacturing industry was 35.7%, down 14.3 percentage points from the previous month. Thus, it reached the lowest PMI value of the whole year, and it was also the period of the most severe epidemic in China.

In March, China's epidemic prevention and control situation continued to improve, production and living order were steadily restored, and enterprises' resumption of work and presentation significantly accelerated. Although, as a result, China purchasing managers' index fell sharply last month on the base, including manufacturing PMI was 52.0%, up 16.3% the previous month, purchasing managers' index rebounded in March to more than a tipping point is the sharp decline after the rebound in February.

Still, more than half of the firms are to return to work, and the production situation has improved since last month. Therefore, it does not represent China's economic operation has returned to normal levels. At the same time, due to the lag of the epidemic and the global spread, the global PMI index gradually fell below 50% from February 2020, reached the lowest 39.8% in April, and slowly recovered from May.

In a crisis like this, cash flow from operating activities is reduced because orders are reduced. SMEs are reluctant to take on additional debt because they will not repay their debts on time. Online business models can provide ideas for firms to increase revenue. According to Baidu Index, the popularity of online office searches reached a peak of 1,367 in February.

First, promoting social media is conducive to SMEs actively displaying their products or businesses and promoting promotional activities. Second, firms can also build online communities to build customer loyalty and effectively understand customers through new tools such as digital marketing to enhance the customer experience. As a result, policies that focus solely on providing government loans are often flawed. Instead, policies must focus on changing the offline business model of many SMEs to embrace online business and adapt to the epidemic situation.

The government should provide necessary online business training to SMEs that need it. For example, according to a report by, the Tianjin Bureau of Commerce has offered online business education for merchants on the WeChat public account “Goutianjin Purchase Tianjin” since February. Such enterprise education and training can be more systematic and refined for different SMEs to provide different content. And information technology offers the necessary support for the online operation of enterprises. The adoption of cloud computing through accounting software is a good example. It reduces the cost of monitoring and accessing the security of financial reports.

In addition, enterprises can use information technology to track inventory and create scientific job management to save valuable business time. Moreover, governments must work on digitizing SMEs and making them part of the ecosystem of the networked economy. Many SMEs will also spontaneously transform the development. Therefore, in addition to encouraging SMEs to develop online businesses, the government should consider providing small grants to SMEs interested in taking the initiative to develop online businesses.

Providing the right incentives is essential to help SMEs adapt to the new realities. Even a small amount of funding can make a big difference in the behavior of small firms and help them increase their ability to generate revenue. The government can also temporarily increase the pre-tax deduction for capital investment, encourage SMEs to invest in technology, and accelerate their technological and online orientation.

The epidemic affects different types of enterprises in other times and degrees of return to work, and March and April 2020 are the time for each industry to return to work and production.

Sixth, generate income for SMEs through government purchases. The Chinese government is a huge buyer of goods and services in the domestic market. In 2018, the government procurement scale nationwide reached 3,586.14 billion yuan, accounting for 4% of China's GDP. In addition, central and local governments and other public infrastructure sectors have become important customers for many businesses through spending on vital public services such as schools, hospitals, and prisons.

Moreover, the government has ample capacity to adjust its procurement budget. However, it is often large firms that benefit from government procurement. If the government adopts only an open tender model where the lowest bidder wins, small firms will not compete with large ones on cost, leading to large firms signing most of the contracts from the public sector. Therefore, the Chinese government will take care of SMEs in government procurement.

In 2018, for example, the total amount of government procurement contracts awarded to small, medium, and micro enterprises reached 2,748.86 billion yuan, accounting for 76.7% of the total amount of government procurement. This proportion can be expanded to support SMEs in the current special circumstances, which are much more difficult than large enterprises. Under the epidemic, the government needs to increase public health services, financial support, and other aspects and increase services and commodities. When conditions permit, the government can hand over these demands to SMEs to produce, providing them with much-needed assistance.

Seventh, timely assessment of policy effects and adjustments. Although the epidemic situation in China has gradually stabilized, the international environment is still not optimistic. To cope with this rapidly changing and unpredictable situation, the government should increase official surveys and set up a database. At the same time, reliable social survey data should be used to make timely and accurate assessments of their supporting behaviors, such as providing commercial loans, and timely adjustments should be made based on this. The government should also investigate SMEs to understand their difficulties and concerns and provide targeted help. In addition, the government can also predict future economic development and changes through various research results to track the effectiveness of policies in the present and future (Brown et al., 2020).

### Long-Term Policy Recommendations

The economic impact of the outbreak may still be a long way off, and this outbreak is not the only external shock in history. Therefore, the government should fundamentally improve the ability of SMEs to deal with external shocks in a long-term way to avoid being strapped for cash every time an accident occurs. To this end, this paper puts forward four policy suggestions that can be implemented in the long term to enhance the crisis resistance of enterprises.

Firstly, promote the digital transformation of SMEs. After realizing the digital transformation, SMEs can easily choose to use the business model of digital design, use information technology and digital network design, and better enhance the value of the enterprise. Furthermore, these digital technologies are conducive to SMEs' better access to customer feedback and timely adjustment to market demand in times of crisis (Schallmo et al., 2018). In addition, digital technology can also establish transparent communication between entrepreneurs and IT experts to prevent corporate fraud (Yadav et al., 2018).

However, digital transformation is not easy and often faces many problems such as lack of funds. Therefore, governments and stakeholders should support the digital transformation of SMEs by effectively addressing the issues of lack of talent, limited knowledge, insufficient technology, insufficient infrastructure, lack of marketing, and lack of digital ecosystem.

Secondly, enterprises should be encouraged to incorporate their ability to resist risks into their construction plans. In the long run, such force majeure events as the epidemic disease are inevitable. Although the life cycle of SMEs is generally short, the construction of anti-risk ability will determine the survival of enterprises in the critical period. The government should encourage enterprises to establish crisis awareness through media publicity and other means, improve labor productivity, reduce capital occupation, pay attention to enterprises' credit, and improve banks' trust in them.

Furthermore, SMEs should make various crisis management plans, collect information in advance to alert against crises, deal with institutional difficulties and manufactured difficulties in a planned way once the problem occurs, and make timely summaries after the crisis to reflect on the deficiencies. After successful crisis management, SMEs can also take this as a case for positive publicity in the media (Lu and Zhang, 2020), improving enterprises' brand awareness and social influence, and providing material for other enterprises to learn.

Thirdly, improve the legal system of SME rescue. At present, there are many laws concerning the daily management of SMEs in China, such as the law on the Development of SMEs, Measures for the Establishment and Management of the Development Fund for SMEs, and Measures for the Management of Credit Guarantee for SMEs. Still, there is a lack of consideration of particular matters, such as the epidemic crisis.

China's legislative and administrative bodies can consider writing the proven epidemic response measures into the aid law for SMEs, legalizing the temporary measures, achieving the effect of benefiting SMEs for the first time after the crisis, and providing timely assistance to SMEs. At the same time, the promulgation of the corresponding laws also reflects the government's sense of distress, which can set an example for enterprises to establish a sense of crisis.

Fourthly, give play to the supporting role of trade associations. With the policy support of local governments, industry associations can play a key role in developing SMEs (Le, 2018). As an organization between government and enterprise, industry association represents civil power and connects enterprise and government. Under the epidemic situation, the resumption of production is the common demand of all SMEs: forming “special market demand” (Lu, 2003). Under the market economy, it is difficult for the government to directly participate in the management of enterprises to provide such “special market demand.” At the same time, the industry association is the best provider of such demand.

Moreover, by integrating industry resources and providing various services for enterprises affiliated, industry associations can unify various economic entities' scattered interests and ultimately guarantee common interests. For example, during the epidemic, China Medical Supplies Association, China Industrial Textiles Association, and other trade associations fully integrated industry resources to ensure the supply of masks and other medical supplies, demonstrating the important role of trade associations.

In addition, as a bridge between the government and enterprises, industry associations can provide grass-roots support for government policies. For example, the China Hemp Textile Industry Association provides a production processing and procurement information platform for member enterprises and governments of industrial agglomerations, effectively promoting the information connection between supply and demand (Ministry of Civil Affairs, 2020).

Finally, the industry association can also play a leading role in enhancing the industry's confidence, for SMEs to tide over the difficulties hit a shot in the heart. Playing industry association role needs to start from long-term. In the stable period of economic development, the government should pay attention to whether the fund budget of the association is sufficient, guarantee its autonomy, establish industry prestige, and play a leading role. Only in this way can the association play its role as the assistant of the government, the hand of the industry, and the helper of the enterprise at all times, can it effectively play its role in emergencies such as the epidemic.

## Conclusion

SMEs have played an irreplaceable role in China's economic development. However, due to the vulnerability of SMEs, COVID-19 is more vulnerable to external shocks such as liquidity crises. The impact is particularly severe for micro-enterprises, start-ups, innovative SMEs, firms based in rural areas and Hubei province, and localized service-oriented SMEs. High-tech and digitalization will reduce the negative impact of the epidemic on SMEs. In addition, government policies can effectively affect the sustainable development of SMEs.

In summary, the main purpose of this study is to provide a detailed list of policy recommendations for policymakers to mitigate better the possible negative effects of the crisis on many small businesses and some suggestions for enterprises to improve their resilience to risk. The reason is that economic recovery depends largely on SMEs' healthy growth, which is also the key issue of China's economic development during the epidemic. This paper argues that such detailed analysis is essential for current economic decisions. Policymakers are often criticized for making bad decisions, especially when public safety and economic development need to be balanced. Therefore, scientific decisions based on adequate analysis and evaluation of objective facts are significant. Given the specific situation in China, controlling the spread of the virus is no longer a priority. While taking advantage of existing conditions to avoid a recurrence of the epidemic crisis, the government should also focus on the sustainable development of SMEs. Therefore, our government needs to formulate a set of sensible and effective measures.

## Data Availability Statement

The data that support the findings of this study are available from the corresponding author upon reasonable request.

## Author Contributions

All authors listed have made a substantial, direct, and intellectual contribution to the work and approved it for publication.

## Funding

This work was supported by the Planning of Philosophy and Social Sciences in Zhejiang Province of China (Grant number: 22NDQN235YB), the Natural Science Foundation in Zhejiang Province (Grant number: LQ20G030013) and HDU (Grant number: KYS145619115).

## Conflict of Interest

The authors declare that the research was conducted in the absence of any commercial or financial relationships that could be construed as a potential conflict of interest.

## Publisher's Note

All claims expressed in this article are solely those of the authors and do not necessarily represent those of their affiliated organizations, or those of the publisher, the editors and the reviewers. Any product that may be evaluated in this article, or claim that may be made by its manufacturer, is not guaranteed or endorsed by the publisher.

## References

[B1] Association of Small and Medium-Sized Enterprises. (2020). Small and Medium-sized Enterprises Development Index. Singapore: Association of Small and Medium-Sized Enterprises.

[B2] Baker S. R. Bloom N. Davis S. J. Terry S. J. (2020). Covid-induced economic uncertainty. Natl. Bureau Econ. Res. 2020:w26983. 10.3386/w26983

[B3] Bouazza A. M. (2015). Establishing the factors affecting the growth of small and medium-sized enterprises in Algeria. Am. Int. J. Social Sci. 4, 101–115.

[B4] Brown R. (2020). Mission-oriented or mission drift? A critical examination of mission-oriented innovation policies. Eur. Plan. Stud. 29, 739–761. 10.1080/09654313.2020.1779189

[B5] Brown R. Lee N. (2019). Strapped for cash? Funding for UK high growth SMEs since the global financial crisis. J. Bus. Res. 99, 37–45. 10.1016/j.jbusres.2019.02.001

[B6] Brown R. Linares-Zegarra J. Wilson J. O. (2018). An Empirical Examination of Alice Borrowers in the UK. Final Report to the Enterprise Research Centre.

[B7] Brown R. Linares-Zegarra J. Wilson J. O. (2019a). Sticking it on plastic: credit card finance and small and medium~sized enterprises in the UK. Reg. Stud. 53, 630–643. 10.1080/00343404.2018.1490016

[B8] Brown R. Linares-Zegarra J. Wilson J. O. (2019b). The impact of (Potential) Brexit on UK SMEs: regional evidence and public policy implications. Reg. Stud. 53, 761–770. 10.1080/00343404.2019.1597267

[B9] Brown R. Rocha A. Cowling M. (2020). Financing entrepreneurship in times of crisis: exploring the impact of Covid-19 on the market for entrepreneurial finance in the UK. Int. Small Bus. J. 38, 380–390. 10.1177/0266242620937464 PMC7342936 38602995

[B10] China.gov.cn. (2020). A Summary of National Government Procurement in 2020. China: The State Council.

[B11] Cowling M. Liu W. Ledger A. (2012). Small business financing in the UK before and during the current financial crisis. Int. Small Bus. J. 30, 778–800. 10.1177/026624261143551627683658

[B12] Cowling M. Liu W. Minniti M. Zhang N. (2016). UK credit and discouragement during the GFC. Small Bus. Econ. 47, 1049–1074. 10.1007/s11187-016-9745-6

[B13] Demirguc-Kunt A. Peria M. S. M. Tressel T. (2020). The global financial crisis and firms' capital structure: Was the impact more severe among SMEs and non-listed firms? J. Corporate Fin. 60, 101–114. 10.1016/j.jcorpfin.2019.101514

[B14] Ding D. (2020). Analysis of the difficulties faced by SMEs in financing under the influence of the new pneumonia epidemic. For. Indus. Sci. Technol. 19, 105–106.

[B15] Doshi H. Kumar P. Yerramilli V. (2018). Uncertainty, capital investment, and risk management. Manag. Sci. 64, 5769–5786. 10.1287/mnsc.2017.2815

[B16] Du L. B. (2021). On the economic recovery and development countermeasures of small and medium-sized enterprises after COVID-19. Times Econ. Trade 18, 27–29.

[B17] Gao J. C. Fan Q. Yang D. G. (2017). Matching effects of corporate movable property financing and macroprudential regulation. Fin. Res. 6, 111–125.

[B18] Government of China. (2020a). The General Office of the State Council issued the “Opinions on the Implementation of the Measures for Strengthening and Stabilizing Employment in Response to the Influences of COVID-19”. Government of China.

[B19] Government of China. (2020b). Increase Financial Support for the Resumption of Work and Production of Micro, Small and Medium-Sized Enterprises. Government of China.

[B20] Haldane A. (2018). The UK's Productivity Problem: Hub No Spokes. Speech at the Academy of Social Sciences Annual Lecture, London.

[B21] Harvie C. Lee B. C. (2005). “Introduction: the role of small and medium-sized enterprises in achieving and sustaining growth and performance,” in Supporting Growth and Performance in East Asia The Role of Small and Medium-Sized Enterprises, eds Harvie C. Lee B. C. (Cheltenham, UK: Edward Elgar Publishing), 3~27.

[B22] Herbane B. (2010). Small business research: time for a crisis-based view. Int. Small Bus. J. 28, 43–64. 10.1177/0266242609350804

[B23] Hubei Provincial Bureau of Statistics. (2020). Economic Situation in the First Quarter of 2020. Hubei: Hubei Provincial Bureau of Statistics.

[B24] Kuckertz A. Brandle L. Gaudig A. Hinderer S. Reyes C. A. M. Prochotta A.. (2020). Start-ups in times of crisis—a rapid response to the COVID-19 pandemic. J. Bus. Ventur. Insights 13:e00169. 10.1016/j.jbvi.2020.e00169

[B25] Le N. N. (2018). Factors Affecting the Development of Industry SMEs in Thai Nguyen Province. Doctoral thesis. Vietnam: Thai Nguyen University.

[B26] Lee N. Brown R. (2017). Innovation, SMEs and the liability of distance: the demand and supply of bank funding in UK peripheral regions. J. Econ. Geogr. 17, 233–260. 10.1093/jeg/lbw011

[B27] Lee N. Sameen H. Cowling M. (2015). Access to finance for innovative SMEs since the financial crisis. Res. Policy 44, 370–380. 10.1016/j.respol.2014.09.008

[B28] Liu Y. (2009). Analysis on the construction of crisis management mechanism of small and medium-sized enterprises. J. Hunan Coll. Finance Econ. 12, 133–134.

[B29] Liu Y. Mou L. (2021). Innovation and transformation of human resource management in small and medium-sized enterprises under normal epidemic prevention and control. Modern Bus. Indus. 42, 72–74.

[B30] Lu L. (2003). Research on the Economic Autonomy of Industry Associations. 1st Ed. Beijing: Law Press.

[B31] Lu M. F. Xu Y. Y. (2020). Study on crisis management mechanism of small and medium-sized enterprises under COVID-19 epidemic situation. J. Luohe Voc. Tech. College 19, 56–60.

[B32] Lu M. F. Zhang H. (2020). Research on the application of blockchain technology in credit economy construction. J. Beijing Voc. Coll. Fin. Trade 2, 11–17.

[B33] Ministry of Civil Affairs. (2020). Industry Associations and Chambers of Commerce Take an Active Part in Epidemic Prevention and Control and Resumption of Work and Production. Ministry of Civil Affairs.

[B34] OECD. (2009). The Impact of the Global Crisis on SME and Entrepreneurship Financing and Policy Responses. Paris: OECD, 1–72.

[B35] OECD. (2020). Coronavirus (COVID-19): SME Policy Responses. Paris: OECD.

[B36] Pal R. Sekh A. A. Kar S. Prasad D. K. (2020). Neural network-based country-wise risk prediction of COVID-19. Appl. Sci. 10, 6448. 10.3390/app10186448

[B37] Robb A. M. Robinson D. T. (2014). The capital structure decisions of new firms. Rev. Fin. Stud. 27, 153–179. 10.1093/rfs/hhs072

[B38] Salimzadeh P. Courvisanos J. Raveendranath R. N. (2013). “Sustainability in Small and Medium-Sized Enterprises in Regional Australia: A Framework of Analysis Annual,” in 26th SEAANZ Conference Proceedings.

[B39] Schallmo D. Williams C.A. Boardman L. (2018). Digital transformation of business models-best practice, enabler, and roadmap. Int. J. Innov. Manag. 21, 17. 10.1142/S136391961740014X

[B40] Wang S. S. Goh J. R. Sornette D. Wang H. Yang E. Y. (2020). Government support for SMES in response to COVID-19: theoretical model using wang transform. Swiss Finance Inst. Res. Paper Ser. 2020, 1–27. 10.2139/ssrn.3608646

[B41] Wilson K. E. (2015). Policy Lessons from Financing Innovative Firms. OECD Science, Technology and Industry Policy Papers, No. 24. Paris: OECD.

[B42] World Bank. (2020). Europe and Central Asia region, International Monetary Fund. The Road to Stability and Prosperity in South-Eastern Europe: A Regional Strategy Paper. Washington, DC: World Bank.

[B43] Wu F. Liu G. J. Guo N. L. Li Z. H. Deng X. Z. (2021). Impact of COVID-19 outbreak on regional economy and industry in China. J. Geogr. 76, 1034–1048. 10.1007/s11442-021-1859-3

[B44] Wu L. Y. Liu Y. Z. (2021). The impact of structural shocks on China's economy and the economic impact of the new crown pneumonia outbreak. Contemp. Fin. Econ. 11, 3–15.

[B45] Xing Y. (2021). Analysis of the development of new retail business models in the post-epidemic era. Trade Show Econ. 23, 95–97.

[B46] Xinhua News Agency. (2019). Ministry of Industry and Information Technology: As of the End of Last Year, the Number of Small and Medium-Sized Enterprises in China has Exceeded 30 Million. Beijing: Xinhua News Agency.

[B47] Xu M. (2020). How to realize innovation development of small and medium-sized enterprises under the influence of COVID-19 epidemic. Guangdong Econ. 7, 67–69.

[B48] Yadav N. Gupta K. Rani L. Rawat D. (2018). “Drivers of sustainability practices and SMEs”: a systematic literature review. Eur. J. Sustain. Dev. 7, 531–544. 10.14207/ejsd.2018.v7n4p531

[B49] Yu L. Tang R. W. Tang Y. C. Yang J. R. Bai Y. X. Jing W. M.. (2011). China's macroeconomic outlook and policy choices. Reform 8, 10–26.

[B50] Zhang J. Chen H. Wu X. (2015). Operation models in the O_2_O supply chain when existing competitive service level. Int. J. e-Serv. Sci. Technol. 8, 279–290. 10.14257/ijunesst.2015.8.9.29

